# Virtual environment to quantify the influence of colour stimuli on the performance of tasks requiring attention

**DOI:** 10.1186/1475-925X-10-74

**Published:** 2011-08-19

**Authors:** Alessandro P Silva, Annie F Frère

**Affiliations:** 1 Núcleo de Pesquisas Tecnológicas, Universidade de Mogi das Cruzes, Mogi das Cruzes, São Paulo, BR

## Abstract

**Background:**

Recent studies indicate that the blue-yellow colour discrimination is impaired in ADHD individuals. However, the relationship between colour and performance has not been investigated. This paper describes the development and the testing of a virtual environment that is capable to quantify the influence of red-green versus blue-yellow colour stimuli on the performance of people in a fun and interactive way, being appropriate for the target audience.

**Methods:**

An interactive computer game based on virtual reality was developed to evaluate the performance of the players.

The game's storyline was based on the story of an old pirate who runs across islands and dangerous seas in search of a lost treasure. Within the game, the player must find and interpret the hints scattered in different scenarios. Two versions of this game were implemented. In the first, hints and information boards were painted using red and green colours. In the second version, these objects were painted using blue and yellow colours. For modelling, texturing, and animating virtual characters and objects the three-dimensional computer graphics tool Blender 3D was used. The textures were created with the GIMP editor to provide visual effects increasing the realism and immersion of the players. The games were tested on 20 non-ADHD volunteers who were divided into two subgroups (A1 and A2) and 20 volunteers with ADHD who were divided into subgroups B1 and B2. Subgroups A1 and B1 used the first version of the game with the hints painted in green-red colors, and subgroups A2 and B2 the second version using the same hints now painted in blue-yellow. The time spent to complete each task of the game was measured.

**Results:**

Data analyzed with ANOVA two-way and posthoc TUKEY LSD showed that the use of blue/yellow instead of green/red colors decreased the game performance of all participants. However, a greater decrease in performance could be observed with ADHD participants where tasks, that require attention, were most affected.

**Conclusions:**

The game proved to be a user-friendly tool capable to detect and quantify the influence of color on the performance of people executing tasks that require attention and showed to be attractive for people with ADHD.

## Background

The attention deficit/hyperactivity disorder (ADHD) is defined as a neuropsychiatric disorder that is characterized essentially by lack of attention, agitation, and impulsiveness, which may lead to poor school performance as well as emotional and relationship difficulties [1]. Lower attention and higher activity can be caused by perinatal morbidity, birth weight, gestational age and socio-economic status at infancy [2].

Virtual reality systems have also been presented as a promising tool in many areas of treatment and rehabilitation [3]. The technological advances associated with cost reductions make the assessment enjoyable and pleasant [4]. In fact, virtual reality, when using dynamic three-dimensional stimuli, can provide a good level of concentration and maintain a high level of involvement and motivation during the entire test [5].

Several authors used virtual reality to evaluate attention through the brain waves of volunteers [6]. In another study, [7] an attention training system was developed, where the volunteers pilot a fighter plane. The aircraft controls as well as their sights are directly related to concentration, i.e., the higher the concentration, the more effective the control and more precise the targeting. However, none of these studies quantified the influence of colours on the attention performance. The attention required in a virtual environment is closely related to the visual effects, and a higher level of immersion can be achieved with a more attractive and engaging content [8]. Advances in computing and new display technologies enabled the development of virtual environments that provide situations similar to those experienced in "real life", enabling detection or treatment of ADHD [6,9]. These benefits were used in the adventure game developed here.

Other studies [10-13] showed that people with ADHD have impairment in the blue-yellow discrimination. In those studies the influence of colours were detected by neuropsychological conventional tests such as Stroop or through questionnaires, but the effect on performance of daily tasks requiring attention has not been studied.

The developed computer game presented in this paper uses a virtual environment to quantify the effect of colour on the performance in accomplishing oriented tasks. It was used to evaluate individuals with ADHD without using frustrating or repetitive tasks that are often annoying to these patients.

Two versions of the same game were implemented. In the first version important objects (hints and information boards) were painted using green/red colours and in the second version, these important objects were painted in blue/yellow.

The developed game resulted in a fun-filled way to relate the perception of some colours to the performance of volunteers with ADHD through a virtual environment.

## Methods

The game "Raiders of the Lost Treasure" was developed using Blender, an open source 3D content creation suite [14]. The use of this software package enabled us to create and manipulate three-dimensional contents, modelling, animation and rendering. The interactivity of the game engine was implemented in logical blocks and programmed using the computer language Python.

The models were constructed by combining and deforming the geometry of basic 3D objects, such as: circles, planes, cubes, spheres, cones, and cylinders. To change the appearance of a surface, the Vertex Paint feature was used, which allowed colours to be applied on the faces of the objects. The texture mapping was performed with the UVMap feature where a map of coordinates (UV coordinates), defines what should be applied on each side of the model. For the animation, the local structure of the bodies was modified to simulate surfaces that contract and stretch in response to external forces (skinning).

### Defining type and characteristics of the game

The computer game "Raiders of the Lost Treasure" was developed to quantify the influence of colour stimuli on the performance of people with ADHD. An adventure game was chosen for being appropriate for children and adults.

Due to the low tolerance of the target audience in relation to failure, the game contains no time limit or virtual life losses. To make the game interesting for ADHD patients, the tasks are shorter and rewards are offered more often than those usually found in commercial adventure games.

The view mode chosen was the first person, so the player can see in more details the scenarios and their characteristics.

Sound hints to indicate the approach of important items were not implemented, making it possible to evaluate only the influence of colour stimuli, as recommended by American Psychological Association [15].

The game is sequential and the tasks follow a logical predefined order,, for example, entering the lake is allowed only after discovering the fishing net.

### Story

Prior to beginning the game, an audio file with an introductory narrative runs and tells the story of an old pirate named Carlos who is interested in finding a lost treasure on a faraway island. When arriving at the island, he discovers that the treasure is located in an abandoned mine's room. To open this room, it is necessary to find a magic key, a mandala that was divided into four pieces, which were hidden in different places on the island. On the way, several coins are available; however, in order to get them it is necessary to avoid poisonous snakes.

Prior to starting the first task, an introductory help text is displayed giving information regarding relevant objects and hints necessary to achieve the goal of the game.

The game was divided into two levels and each level has nine tasks. The first level takes place outdoors, in an island, which favors the player to explore the scene quickly. The second level takes place inside a mine where more attention is needed to meet and overcome the challenges.

**Figure 1:**
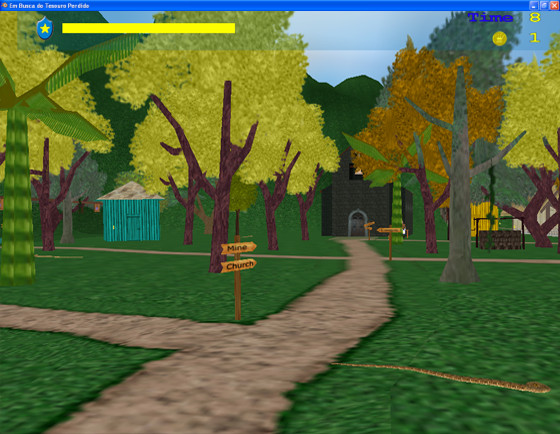
**Scenery of game**. Rendered environment with yellow information boards for user guidance.

In the first level, the first two parts of the mandala are hidden in the island's fishing village and need to be found by the players. In order to help the players to accomplish the task, indication plates and procedure hints were inserted in the environment (Figure 1), as well as various animations were placed in the scenery for concentration and fun In the first level of the game, players would find the sceneries where tasks would be performed by either reading and interpreting the hints, or by exploring the scenery randomly. Even though the accomplishment of tasks is more time consuming when performed without the reading and understanding of the procedure hints, due to the characteristic impulsiveness, hyperactive players would cross the scene quickly, while other volunteers would follow the paths laid out by reading the information.

In the second level, hints help the player to find the two other parts of the mandala. When the search is completed and the mandala is put in place, the treasure room opens. This level was implemented to favour the players who are able to keep high levels of attention and who can remember the location of items placed at strategic points.

### Versions of the game

**Figure 2:**
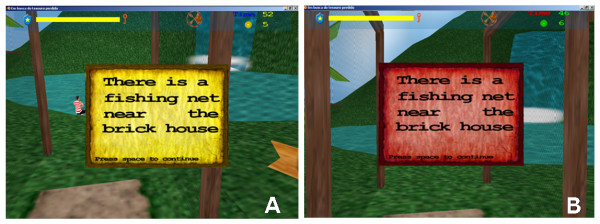
**Example of a hint**. Hint to find the fishing net needed to retrieve the mandala from the bottom of the lake: A) hint in blue-yellow version of the game affecting the performance of volunteers with ADHD; B) hint in red-green version of the game that does not affect the performance of volunteers with ADHD.

**Figure 3:**
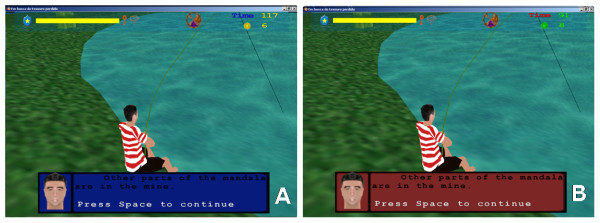
**Instruction provided by a fictional character**. The fictional character telling where to find the other parts of the mandala. A) The yellow-blue version. B) The red-green version.

To quantify the influence of colour perception on the performance of players, two versions of the game were implemented. In the first version, red-green colours are used to display important items in the game such as procedure hints (Figures 2a and 2b), as well as localization hints (information boards) and dialogue screens (Figures 3a and 3b).

In the second version, the colours of these items were changed to the blue-yellow.

Players' performance was evaluated by measuring the time spent to accomplish each task. Although no time limit was imposed to finish the game. Finishing it meant winning, while giving up meant loosing.

### Software engineering

The logical structure of the game was developed using modular programming, which enabled us to reuse several routines during the implementation of the tasks. The scripts were written in a structured manner using the language Python. The software validation of the internal structure of the game was performed using a white box test and a functionality test was performed by a black box test [16].

**Figure 4:**
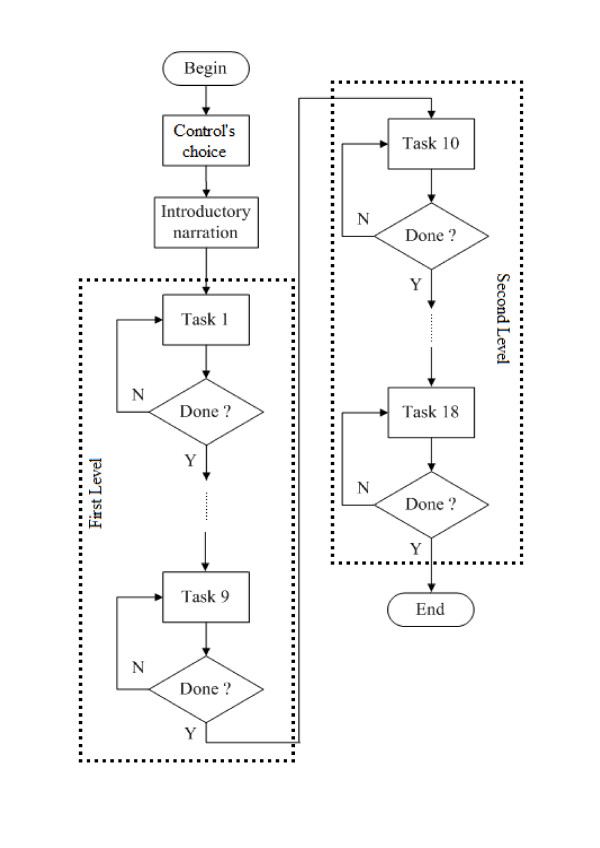
**Logical diagram of the game**. Shows the predetermined logical task sequences of the game.

The tasks are executed in a predefined logical sequential order. Access to the next task is only allowed after completing the previous task. The logical diagram (Figure 4) depicts the sequential task flow of both levels of the game. Initially, before starting the first task, the player has to choose the type of controls he wants to use, and the system then starts the audio narration conceptualising the game. After that the tasks sequence is started and the following sequence of tasks needs to be fulfilled:

Level one:

Task 1- Find the resident who knows the secrets of Treasure Island;

Task 2- Find the trunk where the first part of the mandala is located;

Task 3- Find the key that opens the trunk;

Task 4- Go back to the trunk and catch the first part of the mandala;

Task 5- Find the lake were the second part of the mandala is submerged;

Task 6- Find a fishing net to catch the second part of the mandala;

Task 7- Go back to the lake to catch the second part of the mandala;

Task 8- Locate the mine where the other parts of the mandala resides;

Task 9- Find the axe to break the fence that blocks the mine entrance;

Level two:

Task 10- Find the wizard who knows where the third part of the mandala is located;

Task 11- Find the place indicated by the wizard;

Task 12- Frighten the owl that guards the third part of the mandala;

Task 13- Find the wizard, who then requests the player to locate his lost ring in exchange for the fourth part of the mandala;

Task 14- Find the wizard's ring;

Task 15- Find a piece of meat to capture the tiger that guards the wizard's ring;

Task 16- Returns to the wizard and give him the ring to receive the fourth part of the mandala;

Task 17- Find the treasure room;

Task 18- Finally, insert the completed mandala into the hole on the wall close to the treasure room's door to unlock it.

### Participants

The game developed in this study was approved by the local medical ethics committee. Experimental data was obtained from 17 men and 23 women aged 15 to 25 years old with moderate-to-good experience in computer games. The participants, all students, were selected by evaluating their computer skills through the execution of one introductory level of the commercial computer game "Prince of Persia" [17] with commands and characteristics similar to the game developed for this study. It was up to each user to define which commands to use: the mouse and keyboard, simultaneously or only the keyboard. From the 90 players who accomplished this test in 300 seconds ± 5%, 40 volunteers were recruited, consisting of 20 non-ADHD volunteers (A - control group) and 20 volunteers with ADHD, free of medications (B - experimental group). The classification for ADHD of the 90 volunteers was realized by a psychologist who analysed the data forms filled out by counsellors and teachers of the students, following the procedures of the Scale Adapted Disorder Attention Deficit/Hyperactivity Disorder test [18].

Groups A and B were subdivided into four subgroups of volunteers (A1, A2 and B1, B2). Subgroups A1 and B1 played the first version of the game with a predominance of green-red hints and subgroups A2 and B2 played the second version with predominance of blue/yellow for the objects.

### Usability

The game is normally controlled using a mouse and a keyboard. A second option, selectable during the initialisation of the game, allows the game to be controlled by an alternate script using only a keyboard. In this case the screen pointer can be moved using the line-up, line-down, left and right keys available on a standard keyboard or through the assignment of user selected keys.

### Data analysis

The performance of the players was measured, recording the elapsed time in seconds needed to perform each task. At the end of each task, a script stores the value in a variable and assigns 0 to the time counter initiating next task. At the end of the game these values, the name of the volunteer, as well as the day on which the test was performed, were stored in a log file.

The data analysis was performed using MatLab ^®^. First, Tm_g, k _was calculated, defined as the average time needed by the subgroup "g" to execute a task "k" and was obtained using equation (1) where T_g, v, k _is the time needed by volunteer "v" of the subgroup "g" to complete task "k". As stated before, the volunteers "v" were divided in four subgroups "g", (A1, A2, B1 and B2) and the game consisted of 18 tasks "k", 9 in each level of the game.

(1) $$Tm_{g,k}=\frac{\sum_{\text{v}=1}^{10}{\text{T}}_{\text{g,k,v}}}{10}$$

The average execution time "TmF1_g_" needed by subgroup "g" to complete the first-level of the game (tasks 1 to 9) as well as the average time needed to complete the second-level "TmF2 _g_" (task 10 to 18), are calculated using equations (2) and (3).

(2) $$TmF1_{g}=\sum_{\text{k}=1}^{9}\text{T}{\text{m}}_{\text{g,k}}$$

(3) $$TmF2_{g}=\sum_{\text{k}=10}^{18}\text{T}{\text{m}}_{\text{g,k}}$$

The average time needed to complete the full game "Tmt_g_" for each subgroup "g" was calculated using equation (4);

(4) $$Tmt_{g}=TmF1_{g}+TmF2_{g}$$

The authors also calculated the total time needed to complete the first-level "TmtF1" by all four members of subgroup "g".

(5) $$TmtF1=\sum_{g=1}^{4}TmF1_{g}$$

Similarly, the second-level total time "TmtF2" was calculated and obtained by the expression:

(6) $$\text{TmtF}2=\sum_{\text{g}=1}^{4}\text{TmF}2_{\text{g}}$$

The obtained results were analysed with the D'Agostino's test to prove that the distribution was normal. The Two-way ANOVA test was used to detect if significant difference exists between subgroups and the posthoc Tukey - LSD (least significant difference) test was used to define which subgroup was different from the remaining subgroups.

## Results

### First level - external scenery

**Figure 5:**
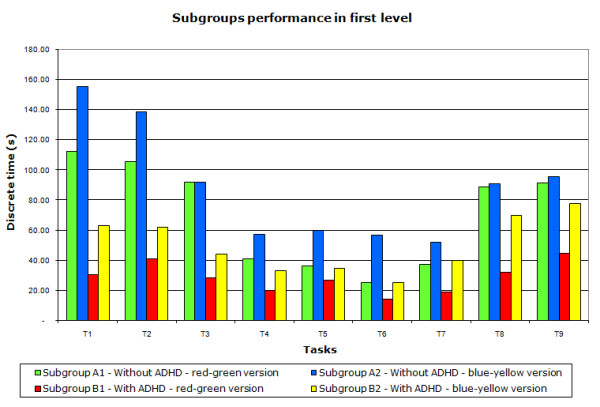
**Average time for each task of the first level**. The average time accomplished by each of the four subgroups in tasks T1 to T9 showing that players with ADHD (subgroups B1 and B2), who explore the scene quickly, performed the tasks faster than the other players (subgroups A1 and A2).

The analysis of the results showed that the ADHD subgroups (B1 and B2) had the best performance during the first level of the game (tasks T1 to T9) as they explored the scene quickly performing the tasks in less time (Figure 5 and Table 1).

**Table 1:** Subgroup average time in the first level "TmF1_g_"

Versions	Subgroups	Time (s)	Standard deviation
**green-red**	A1 - without-ADHD	629	34
	B1 - ADHD	256	10

**blue-yellow**	A2 - without -ADHD	797	37
	B2 - ADHD	449	18

Subgroup B1 completed the first level of the game 43% faster than subgroup B2 and 59.30% faster than the subgroup A1, while subgroup A1 was 21% faster than A2. Statistical analysis two-way ANOVA with p < 0.05 and posthoc TUKEY - LSD (least significant difference) showed a significant difference between all subgroups.

### Second level - Internal scenery

**Figure 6:**
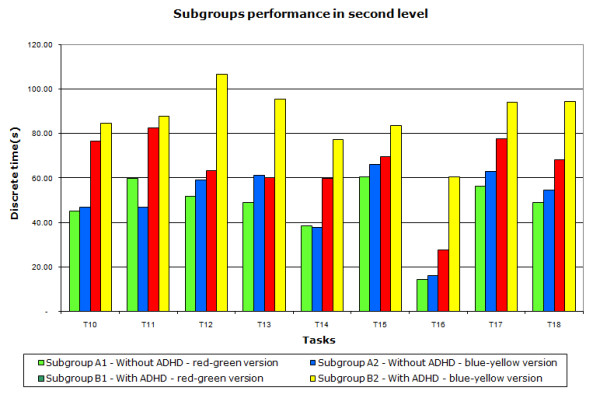
**Average time for each discrete task of the second level**. The average time accomplished by each of the four subgroups in tasks T10 to T18 showing that players without ADHD (subgroups A1 and A2), capable to keep attention, performed the tasks in shorter time than the other players (subgroups B1 and B2).

In the second level (task T10 to T18), where the activities required more attention, players without ADHD (A1 and A2) completed the tasks faster than those with ADHD (B1 and B2). A1 was 27.5% faster than B1; A2 was 42.5% faster than B2; A1 was 6% faster than A2 and B1 was 25% faster than B2 (Figure 6 and Table 2). Task T12 is an example of a task that requires attention where the player must remember the existence of a torch that illuminates the ladder and combine this information with the hint "Owls are afraid of fire", which the non-ADHD volunteers (A1 and A2) performed quickly. However, people with ADHD (B1 and B2) did not notice the torch.

**Table 2:** Subgroup average times for the second level "TmF2_g_"

Versions	Subgroups	Time (s)	Standard deviation
**green-red**	A1 - without- ADHD	424	14
	B1 - ADHD	585	16

**blue-yellow**	A2 - without- ADHD	451	16
	B2 - ADHD	784	13

For the second level of the game, the two-way ANOVA test with p < 0.05 in conjunction with posthoc Tukey - LSD (least significant difference) showed that subgroup B1 was significantly different from B2. However, subgroups A1 and A2 showed no significant difference. These results show that change of colours affect the performance for tasks requiring attention for ADHD subgroups.

### Full Game

**Figure 7:**
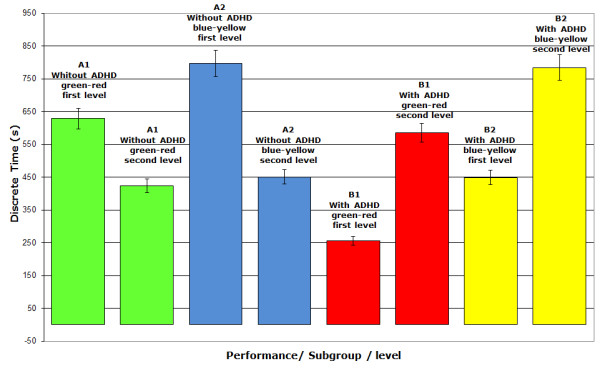
**Average time spent by each subgroup at each level**. The average time of each subgroup at each level indicates that the subgroups B1 and B2 (with ADHD) completed the tasks of the first level faster than the other subgroups (non-ADHD). In the second level a reversal in performance can be observed between these groups favoring those who are more capable to keep attention.

The performance of each subgroup in each level of the game is depicted in Figure 7 and illustrates the influence of color on the performance of tasks needing attention.

**Figure 8:**
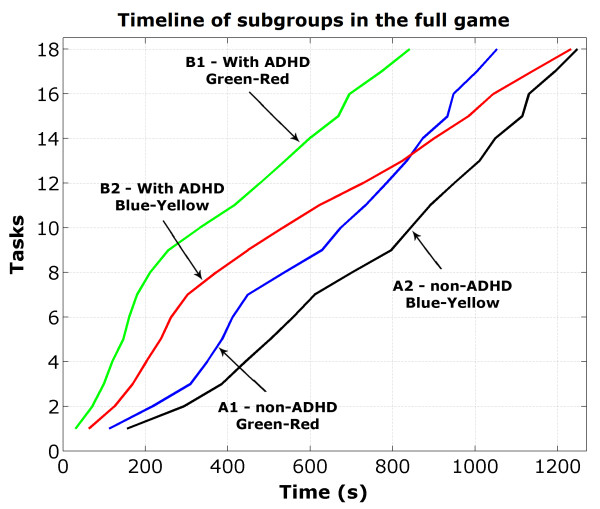
**Performance of each subgroup in the full game**. The evolution of the performance of each subgroup is represented by timelines covering the entire game.

The timeline showing the behavior of each subgroup during the execution of the game is shown in Figure 8.

The first level favors the group with ADHD (B1, B2) and the second level the non-ADHD group (A1, A2). If the full game doesn't favor one group over the other, the sum of the average times spent to complete the first level by all the participants should be similar to that of the second level.

The results described in table 3 show a difference of 5%. The one-way ANOVA test indicates that no significant difference exists between the times spent to complete level 1 and level 2 proving that none of the groups were favored in the game.

**Table 3:** Total time for each level

Levels	Time (s)
**First level -TmtF1**	2130
**Second level - TmtF2**	2244

The data of the game presented in table 4 shows the comparison of the average times and respective percentages spent by people with and without ADHD for both levels of each version of the game (Green-Red and Blue-Yellow). From the data it is clear that a substantial difference exists between the execution times for people with and without ADHD.

**Table 4:** Average times and percentages with/without ADHD

	Without ADHD			With ADHD			Difference
**Used Colours**	**Average time spend**	**Percentage of game time spent**	**Disbalance in favour of level 1 in %**	**Average time spend**	**Percentage of game time spent**	**Disbalance in favour of level 1 in %**	

**Green-red**							
Game level 1	629	59,7	19,5	256	30,4	-39,1	58,6
Game level 2	424	40,3		585	69,6		
**Blue-Yellow**							
Game level 1	797	63,9	27,7	449	36,4	-27,3	55
Game level 2	451	36,1		786	63,6		

## Discussion

The graphical tool Blender 3D provided an interface that enhanced the realism and the immersion level in the game story, fulfilling the established requirements for this research such as designing a game attractive to people with ADHD. To encourage the immersion level for these persons, we inserted detailed textures and several continuous animations in the virtual environment [8]. However, in order to avoid large graphic processing efforts during execution we started the animation of all objects in the same timeline. This resource also helped in the verification of actions sequences.

The game was divided in two levels: the first being an external environment taking place on an island where several items, needed to complete the tasks, were spread. This level favours players with ADHD who tend to explore the scene very quickly [15].

The second level takes place inside a mine. During this phase more attention is needed to find the items and to overcome the challenges. To do so, the players need to remember the previous travelled paths, which was easy for the volunteers without ADHD (A1 and A2). On the other hand, volunteers with ADHD (B1 and B2) were frequently lost during this phase, spending more time to conclude their tasks.

Players with ADHD (B1 and B2) performed better during the first phase, while those without these characteristics (A1 and A2) proved to be more efficient during the second phase. Analysing the time needed to complete the whole game, we observed that the game did not favoured any of these groups.

Moreover, studies [10,12,13] showed that people with ADHD have an impaired colour discrimination in the blue-yellow pathway, therefore, we developed two versions of the game application. In the first version, objects with hints, screen dialogues and information signs were represented using predominantly red-green colours. In the second version, blue-yellow were the predominant colours used to display these objects. The results showed that the subgroups (A1 and B1) using the green-red version of the game performed better than subgroups (A2 and B2). However, this effect was observed to be much larger in individuals with ADHD.

To accomplish this research, we developed a virtual environment offering increased immersion and interest for people with ADHD as also shown in other studies [6,7,19]. The success of this approach was confirmed in our study since all participants reached the end of the game successfully.

Many studies using virtual environments have been carried out by other research groups in order to: change the scale of visual skills [20]; perform cognitive training [19]; enhance attention [7]; evaluate attention [6] and to aid in ADHD diagnosis [21].

Other studies analysed the changes in cognitive processing of children with ADHD [22] as well as colour discrimination impairments in people with ADHD [10-13].

This particular colour impairment was analysed in this study using the developed game providing new contributions to the field. As a matter of fact, this playful resource allowed us to control the lack of motivation which can harm the performance of ADHD volunteers in task-oriented activities. This behaviour can influence experimental results and was therefore addressed up-front in our research.

Previous studies [10,12,13], applying the Stroop test, demonstrated that subjects with ADHD committed more errors than non-ADHD subjects in discriminating colours. However, researchers [10] suggested the use of another method to clarify color vision deficits in ADHD.

The tests presented in this paper suggest a new method to analyse and quantify the dependence of colours on the performance of activities related to colour using tasks closer to those encountered in daily activities. In our test the participants were submitted to tasks that required attention, their performance was determined using the time spent to complete these tasks successfully.

Many variables can affect the information collected in computer games such as: the ability in the use of command interface and the lack of collaboration from participants. In our research, through the use of a virtual environment that provides a playful and immersive mechanism, we were able to evaluate the performance of ADHD subjects without the influence of perceptions of boring and repetitive tasks. This, coupled to a selection process of volunteers having similar computer skills, enabled us to obtain standard deviations of less than 5% for task execution times within the same group (Tables 1 and 2). Therefore we believe that our method was not susceptible to uncontrolled variables.

ADHD can generate many social and behavioural problems including low learning capabilities. Studying the influence of colours used in conveying information can be an important aid to reduce the impact of this disorder and its implications on individuals affected by ADHD.

## Conclusions

This study confirmed the results obtained by other researchers [10-13], that people with ADHD are influenced by colours. We also showed that these colours affect the performance of tasks that simulate daily activities requiring attention.

The playful game, developed for this purpose, proved to be a new useful and attractive tool to perform this evaluation, allowing the acquisition of quantitative data. It enabled us to compare the behaviour of persons with and without ADHD. The game can be easily adapted to perform other evaluations, such as, text change, tasks change, or the introduction of sound. The collected data shows also a substantial difference in performance between level 1 and 2 of the game for people with and without ADHD. Future work is needed to analyse if the developed virtual environment can contribute in a more ludic and friendly way to diagnose ADHD.

## Competing interests

The authors declare that they have no competing interests.

## Authors' contributions

APS and AFF participated in the concept and development of the game. APS and AFF also participated in the acquisition, analysis, and interpretation of the data. All authors revised and approved the current version of the manuscript.
